# Cellular Type Is a Major Determinant of R-Loop Genomic Distribution

**DOI:** 10.32607/actanaturae.27833

**Published:** 2026

**Authors:** K. Yu. Oleynikova, N. A. Zhigalova, A. P. Hutchins, A. S. Ruzov

**Affiliations:** Institute of Bioengineering, Research Center of Biotechnology, Russian Academy of Sciences, Moscow, 117312 Russia; Infochemistry Scientific Center, ITMO University, St. Petersburg, 197101 Russia; Department of Systems Biology, School of Life Sciences, Southern University of Science and Technology, Shenzhen, 518055 China

**Keywords:** R-loops, DNA:RNA hybrids, DNA-RNA Immunoprecipitation (DRIP), cellular differentiation, hPSCs, HAP1 cells

## Abstract

R-loops that contain a DNA:RNA hybrid and unpaired single-stranded DNA are important determinants of normal cell physiology and of the pathogenesis of numerous diseases. Although several different approaches to R-loop mapping in the genome have been developed, these techniques can produce conflicting results. In order to assess their robustness, a recent study by Chedin et al. compared the R-loop genomic distribution assessed using different methods in normal and cancer cell lines. Importantly, that study assumed a high degree of similarity between R-loop genomic distributions across different cellular types. Here, we compared DRIP datasets produced using the same protocol in different cell lines to show that only 26% of R-loop peaks are shared between chronic myeloid leukemia-derived HAP1 cells and human pluripotent stem cells. Meanwhile, HAP1-derived double knockout cell lines are characterized by much higher fractions of R-loop peaks that are identical both to each other (most of them) and to the R-loop peaks of their parental line (71 and 55%). We conclude that cellular type represents a major determinant of R-loop genomic distribution and, therefore, that only a systematic comparison of a large array of various cell/tissue type-derived R-loop datasets may address the inconsistencies between different R-loop mapping techniques.

## INTRODUCTION

R-loops that contain a DNA:RNA hybrid and unpaired single-stranded DNA are abundant in the genome and can be involved in the regulation of a broad range of biological processes, such as transcription termination, DNA repair, telomere homeostasis, and immunoglobulin class-switch recombination [1]. Meanwhile, irregular or pathological R-loops can disrupt transcription and replication, causing accumulation of DNA double-strand breaks and thus becoming a major source of genetic stress and instability in mammalian cells [1]. Taking into account the association between genomic instability and oncogenic transformation, the interest in the regulation of the R-loop distribution across various systems has led to the development of experimental methods for genome-wide R-loop mapping [2]. Some of these methods are based on antibodies specific to RNA:DNA hybrids (the S9.6 antibodies) [3]. These techniques include DNA:RNA immunoprecipitation (DRIP), with its numerous variants (DRIPc-seq; ssDRIP-seq, etc.), and R-loop cleavage under targets & tagmentation [4, 5]. Other methods are based on either mapping single-stranded DNA within R-loops using bisulfite [6] or the application of RNase H1, an enzyme responsible for the recognition and cleavage of RNA:DNA hybrids [7]. Importantly, R-loop sets obtained using different (and sometimes similar) methods on different cell lines can often be substantially different [8].

In order to resolve these contradictions, Chedin et al. compared the datasets on R-loop genomic distribution obtained for various cell lines using different methods and assessed their degree of similarity [8]. That study does not fully consider the cellular types used to obtain the datasets. The implicit assumption was that there exists a fundamental similarity in R-loop distribution in housekeeping genes across different cellular types. Nonetheless, the validity of this assumption is unclear. Chedin et al. neither evaluated nor discussed the potential extent of differences in the R-loop genomic distribution in various cellular systems such as human pluripotent stem cells (hPSCs) and tumor cell lines (U2OS or HeLa). Instead, they compared the signal coverage profiles from corresponding DRIP experiments performed using different cellular types to identify the datasets considered “discordant” [8]. We believe that this approach, which implicitly presumes similarity in the R-loop genomic distribution across cell lines of different origins, has the potential to distort the interpretation of experimental data.

## EXPERIMENTAL

**Cell lines and cell culture, DRIP, and high-throughput sequencing library preparation**

HNRNPA2B1 KO and YTHDF2 KO cells, as well as isogenic parental wild-type HAP1 cells (Horizon Discovery, # HZGHC007378c010, # HZGHC006678c001, and # C631), were cultured in the DMEM/F12 medium (Gibco Life Technologies, USA # 11320033). DNA–RNA immunoprecipitation (DRIP) was conducted in compliance with the previously published procedure, including control over DRIP signal specificity by treating samples with RNase H [9]. The genomic libraries were constructed using a NEBNext Ultra II DNA Library Prep Kit for Illumina (NEB, USA # E7645) according to the manufacturer’s protocol.

**Bioinformatics analysis of the DRIP-seq data**

Datasets on HAP1 (WT, HNRNPA2B1 KO and YTHDF2 KO) and the previously published dataset on hPSC (PRJNA474076) were analyzed simultaneously. The peaks were identified using a MACS2 peak caller. Broad peaks were detected using the following parameters: −format BAM -g hs −keepdup all. The consensus peaks were identified using the bedtools intersect tool with the standard parameters to ensure minimal input peak intersection (-f 1E-9 -F 1E-9). A detailed description of bioinformatics analysis is available online at https://github. com/katerinaoleynikova/human_samples_paper_25.

## RESULTS AND DISCUSSION

**Fig. 1:**
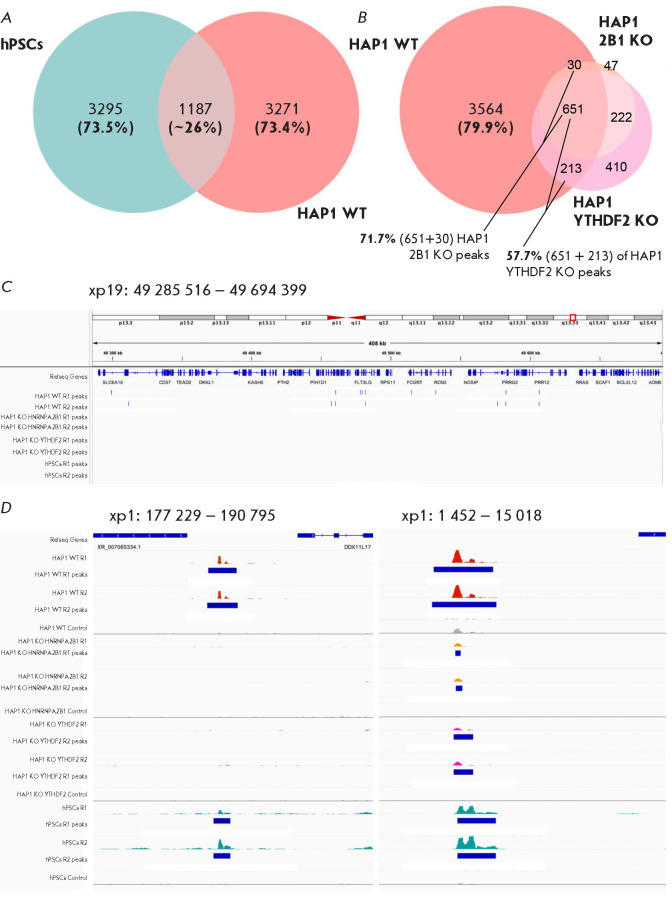
Cellular type is a major determinant of the R-loop genomic distribution. (*A*) Venn diagrams showing the overlaps between the R-loop peak datasets obtained using DRIP experiments in hPSC and wild-type HAP1 (HAP1 WT) cells, as well as (*B*) wild-type HAP1cells and HAP1 cells with genetic knockouts of the *YTHDF2 *(HAP1 YTHDF2 KO) and *HNRNPA2B1 *(HAP1 2B1 KO) genes. The numbers of R-loop peaks in each category are indicated on the diagrams. (*C*) Genome browser view of the distribution of R-loop peaks (shown as blue vertical dashes) in the datasets generated in our DRIP experiments on the aforementioned cell lines over the region centered around the *RPL13A *housekeeping gene used by Chedin et al. as the “gold standard” for dataset comparison. (*D*) Genome browser views of the signal profiles of our R-loop DRIP datasets alongside the control input samples at two representative genomic loci. The locations of the R-loop peaks are shown with blue rectangles

In order to assess the degree of similarity in the R-loop genomic distribution across cells of different origin, we compared DRIP datasets generated using our previously published protocol [9] in three different cellular models: human pluripotent stem cells (hPSCs), wild-type HAP1 (HAP1 WT) cells derived from the KBM-7 chronic myeloid leukemia cell line [10], and two isogenic HAP1 cell lines with *YTHDF2* or *HNRNPA2B1 *gene knockout generated via gene editing (HAP1 YTHDF2 KO and HAP1 2B1 KO cell lines, respectively). The genomic regions enriched in R-loops in these cell lines were identified by DRIPsequencing; the reads were mapped to the human genome and R-loop peaks were identified. Next, consensus peaks for each sample were identified by comparing two corresponding replicates. We demonstrated that, although a similar number of consensus peaks were identified for hPSCs and wild-type HAP1 cells (4,482 and 4,458, respectively), only 1,187 (~26%) peaks were common to both cell lines (*Fig. 1A*). Interestingly, while *YTHDF2 *and *HNRNPA2B1 *gene knockout substantially reduced the total number of peaks (1,496 and 950, respectively) compared to wild-type HAP1 cells, the HAP1 YTHDF2 KO and HAP1 2B1 KO datasets were similar to each other. The vast majority of peaks in HAP1 2B1 KO cells (873 out of 950) overlapped with those identified in HAP1 YTHDF2 KO cells (*Fig. 1B*). Furthermore, 71% (681) of the peaks identified in HAP1 2B1 KO cells and 55% (864) of the peaks in HAP1 YTHDF2 KO cells were identical to the peaks of R-loops detected in their parental HAP1 WT cell line (*Fig. 1B*). The genomic region surrounding the *RPL13A *housekeeping gene, which was used by Chedin et al. for dataset comparison and called “the gold-standard region,” contained only peaks from wild-type HAP1 cells, but not the peaks from R-loops derived from hPSCs or the two other knockout cell lines tested in our study (*Fig. 1C*). We also identified other loci where R-loop peaks were present across all four tested cell lines (*Fig. 1D*, left panel). However, we believe that in order to refer to any genomic regions as “gold-standard” ones, datasets from additional cell lines of diverse origins need to be obtained, since the R-loop genomic distribution appears to be highly cellular-type specific. Hence, we demonstrated that R-loop sets obtained in cell lines of different origins using the same method differ substantially and that only 25% of R-loop peaks coincide between hPSCs and the HAP1 line derived from chronic myeloid leukemia cells. Meanwhile, the R-loop distribution in *YTHDF2 *and *HNRNPA2B1 *knockout HAP1 cells and in wild-type HAP1 cells is apparently more similar for these two cellular types compared to their distribution in hPSCs. Since the recently identified factors involved in R-loop regulation include the chromatin structure [10], nucleosome positioning [11], and RNA modifications [9], all varying significantly across cellular types, these findings are not particularly un expected [12]. Importantly, the interpretation of our results and the conclusions derived from them are confined to the selected models. Therefore, datasets from additional differentiated and tumor cell lines of different histogeneses need to be analyzed to ensure a more robust generalization.

## CONCLUSIONS

Hence, we infer that the R-loop genomic distribution is cellular-type specific. Therefore, only the systematic and standardized comparison of a substantial array of R-loop genomic distribution datasets derived from different cellular types can resolve the discrepancies between the experimental results obtained using different R-loop mapping techniques.

## Abbreviations

DRIP: DNA-RNA immunoprecipitation
HNRNPA2B1: heterogeneous nuclear ribonucleoprotein A2/B1
hPSC: human pluripotent stem cells
